# Erucic Acid (22:1n-9) in Fish Feed, Farmed, and Wild Fish and Seafood Products

**DOI:** 10.3390/nu10101443

**Published:** 2018-10-05

**Authors:** Nini H. Sissener, Robin Ørnsrud, Monica Sanden, Livar Frøyland, Sofie Remø, Anne-Katrine Lundebye

**Affiliations:** Institute of Marine Research, PO box 1870 Nordnes, N-5817 Bergen, Norway; ror@hi.no (R.Ø.); msa@hi.no (M.S.); lfr@hi.no (L.F.); sre@hi.no (S.R.); anne-katrine.lundebye@hi.no (A.-K.L.)

**Keywords:** erucic acid, fish, seafood

## Abstract

The European Food Safety Authority (EFSA) published a risk assessment of erucic acid (22:1n-9) in 2016, establishing a Tolerable Daily Intake (TDI) for humans of 7 mg kg^−1^ body weight per day. This report largely excluded the contribution of erucic acid from fish and seafood, due to this fatty acid often not being reported separately in seafood. The Institute of Marine Research (IMR) in Norway analyzes erucic acid and has accumulated extensive data from analyses of fish feeds, farmed and wild fish, and seafood products. Our data show that rapeseed oil (low erucic acid varieties) and fish oil are the main sources of erucic acid in feed for farmed fish. Erucic acid content increases with total fat content, both in farmed and wild fish, and it is particularly high in fish liver, fish oil, and oily fish, such as mackerel. We show that the current TDI could be exceeded with a 200 g meal of mackerel, as at the maximum concentration analyzed, such a meal would contribute 143% to the TDI of a 60 kg person. These data cover a current knowledge gap in the scientific literature regarding the content of erucic acid in fish and seafood.

## 1. Introduction

The long-chain fatty acid erucic acid (22:1n-9) is a naturally occurring fatty acid, found in high concentrations in seeds of the family Brassicaceae, such as rapeseed and mustard seed. Even though natural forms of rapeseed contain high levels of erucic acid (usually more than 40% of the total fatty acids), commercially bred rapeseed cultivars today (also known as canola) typically have levels below 0.5% of total fatty acids [1]. High erucic acid cultivars are still grown for industrial, non-food purposes, while mustard oil that is sold for human consumption still may have a high content of erucic acid (up to ~50%) [2]. Low concentrations of erucic acid are naturally present in other food sources, such as fish.

The heart is the target organ for adverse effects of exposure to high concentrations of erucic acid, which may lead to lipidosis (accumulation of triacylglycerol as lipid droplets) in the heart muscle, reduced contractility, and eventually tissue damage [3]. This condition has never been documented in humans, but in both experimental and production animals, such as rats, pigs, and chicken [4]. The cause appears to be poor mitochondrial beta-oxidation of this fatty acid, especially in the heart, resulting in an accumulation of erucic acid in neutral lipid droplets. There is primarily an increase in triacylglycerol, while the levels of phospholipids and cholesterol remain relatively constant [5].

The European Food Safety Authority (EFSA) published a risk assessment of erucic acid in feed and food in 2016 [4], establishing a Tolerable Daily Intake (TDI) for humans of 7 mg/kg body weight per day based on the occurrence of cardiac lipidosis in experimental animals. The EFSA report largely excluded the contribution of erucic acid from seafood, on the following grounds: *“Besides the occurrence in oil seeds, erucic acid also occurs naturally in fish and seafood. These food groups mainly contain cetoleic acid (22:1n-11), which is usually accompanied by minor proportions of erucic acid* [6]*. It is also of importance that the database of the Max Rubner Institut provides a single value for 22:1 fatty acids, with no distinction between erucic acid (22:1n-9) and cetoleic acid (22:1n-11) or between the cis and trans isomers. Similarly, the US Department of Agriculture database does not differentiate between erucic acid and cetoleic acid, and it reports cis 22:1 as the sum of both 22:1n-9 and 22:1n-11. Therefore, information from these databases has not been included”.* Clearly, there is a knowledge gap regarding the occurrence of erucic acid in fish and seafood.

Erucic acid (22:1 n-9) and cetoleic acid (22:1 n-11) are quantified separately at the Institute of Marine Research (IMR) in Norway, with an accredited method that obtains good results in proficiency testing (ring trials). Validity tests and parameter controls are performed on a daily routine basis and our quality control systems are audited annually by Norwegian Accreditation, which is the national signatory of the European co-operation for Accreditation Multilateral Agreement (EA MLA). Through national surveillance programs of fish feed, farmed fish, wild caught fish, and commercial seafood products, our institute has extensive data on the fatty acid composition of these products, including the content of erucic acid. While some of these data are available in our seafood database [7], data on erucic acid have never been compiled for different seafood products and have not been published in a scientific journal. Furthermore, we have unpublished data on the metabolism of erucic acid in farmed fish from feeding trials with Atlantic salmon (where the main focus of previous publications has been on other fatty acids). This paper aims to fill current knowledge gaps in the scientific literature on erucic acid in seafood, by giving an overview of data on the occurrence of erucic acid in fish and seafood, and how this fatty acid is metabolized in farmed fish. The data are discussed in the context of fish and human health.

## 2. Materials and Methods

### 2.1. Feed, Fish and Seafood Samples

Data for this paper was compiled from analyses that were conducted in surveillance programs on fish feed, farmed fish, wild fish, and seafood products performed on behalf of the Norwegian Food Safety Authority. Samples of fish feed and fish feed ingredients were sampled by the Norwegian Food Safety Authority from Norwegian feed producers or at fish farms, representative of fish feed production in Norway. Before analysis, feed samples were prepared using standardized methods for the official control of feed. Samples of farmed fish were collected by official inspectors from the Norwegian Food Safety Authority from all fish-producing regions in Norway. The sampling was randomized with regard to season and geographical location. Wild fish and seafood samples were also collected through national surveillance programs in Norway.

### 2.2. Method of Fatty Acid Analysis

**Figure 1:**
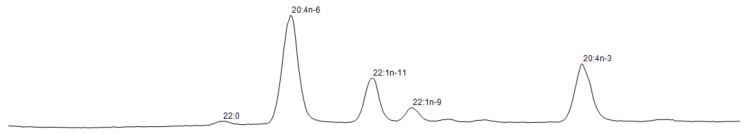
Chromatogram showing the separation of cetoleic acid (22:1n-11) and erucic acid (22:1n-9) in the fatty acid analysis.

Fatty acids were analyzed by an accredited method. Briefly, lipids from the samples were extracted in chloroform/methanol (2:1, *v*/*v*), according to the method of Folch et al. [8]. The extracted lipids were filtered, saponified, and fatty acid methyl esters were prepared by boron trifluoride (12% BF_3_ in methanol), as described previously [9]. Methyl esters were separated on a Thermo Finnegan Trace 2000 GC equipped with a fused silica capillary column (CP-sil 88; Chrompak Ltd., Middelburg, The Netherlands). The temperature programming was 60 °C for 1 min, 160 °C for 28 min, 190 °C for 17 min, and finally, 220 °C for 10 min, with all temperature ramps being 25 °C per min. Individual methyl esters were identified by retention time in comparison to standard mixtures of methyl esters (Nu-Chek, Elysian, Waterville, MN, USA). All of the samples were integrated using the software Chromeleon^®^, connected to the gas liquid chromatograph. Amount of fatty acid per gram sample was calculated using 19:0 methyl-ester as an internal standard. The separation of 22:1n-9 and 22:1n-11 is shown in Figure 1.

### 2.3. Feeding Trial with Atlantic Salmon

Data are from a published feeding trial with Atlantic salmon [10], where data specifically on erucic acid were not included. Fatty acid analysis was conducted, as described above, in some cases with prior separation of the neutral and polar lipid fraction, as described by Sissener et al. [11]. Yttrium oxide for digestibility calculations was analysed in freeze dried faeces and feed samples by ICP-MS (inductively coupled plasma mass spectrometry) [12]. Digestibility was calculated as follows:

Apparent digestibility coefficient = 100 − (% yttrium in feed ÷ % yttrium in feces) × (% nutrient in feces ÷ % nutrient in feed) × 100)(1)

Retention of erucic acid in whole fish during the experiment was calculated as follows:

Nutrient retention = (final biomass × final nutrient concentration − initial biomass × initial nutrient concentration) × 100 ÷ (Total feed intake × nutrient concentration in feed). (2)

Linear regression analyses were conducted in GraphPad Prism 6.0 (Graphpad Software Inc., La Jolla, CA, USA).

## 3. Results

### 3.1. Content of Erucic Acid in Fish Feed Ingredients and Fish Feed

Erucic acid as a percentage of total fatty acids analyzed in fish feed ingredients in the period 2004–2008 and in fish feed from 2006, 2008, and 2012–2016 is given in Table 1. Erucic acid is present in several feed ingredients, with the highest maximum value being found in rapeseed oil (canola) at 3.2% of fatty acids, and secondly in fish oil with 2.5%. The mean values are, however, similar in fish oil, rapeseed oil, and fish silage (where the lipid fraction naturally would be similar to fish oil).

Additionally, the concentration of erucic acid in mg/100 g in fish feeds from 2012–2016 is given in Table 2, while quantitative data are not available for the feed ingredients or the feed samples that were collected before 2012.

### 3.2. Transfer from Feed to Fish

**Figure 2:**
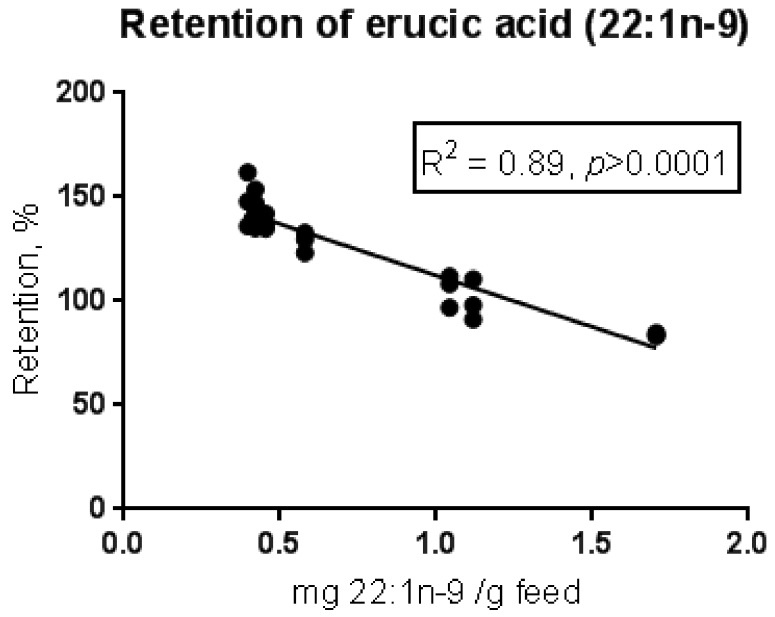
Retention (the amount of erucic acid accumulated in the fish during the feeding period in percentage of the amount eaten) of erucic acid in whole fish in relation to dietary content of erucic acid, in a feeding trial with Atlantic salmon [10] given eight experimental feeds ranging from 40–170 mg erucic acid/100 g feed. The adjusted *R*-value and the *p*-value for a linear regression model are given.

The digestibility of erucic acid was calculated from a previously published feeding trial with Atlantic salmon [10], with an average value of 98.1%. The digestibility was not affected by the content of erucic acid in the eight experimental feeds, which varied from 40–170 mg/100 g feed. The water temperature was 12 °C and the digestibility of erucic acid was similar to other unsaturated fatty acids of similar chain length. Fatty acid retention in salmon (the amount of a fatty acid accumulated in the fish, in percentage of the amount of the fatty acid consumed from the feed) was also calculated. The retention varied among the dietary groups, and it displayed a clear decline with increasing dietary erucic acid concentration (see Figure 2).

**Figure 3:**
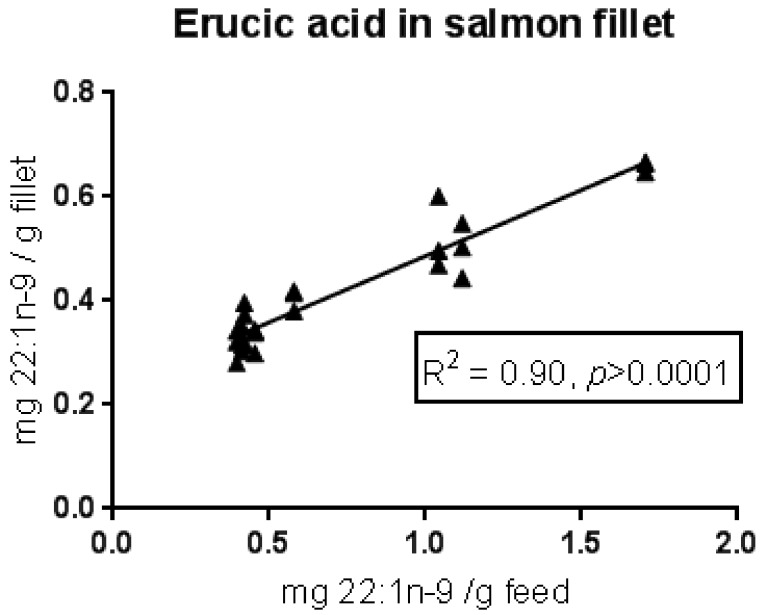
Content of erucic acid (22:1n-9) in salmon fillet in relation to dietary content of erucic acid, in a feeding trial with Atlantic salmon [10] given eight experimental feeds ranging from 40–170 mg erucic acid/100 g feed. The adjusted *R*-value and the *p*-value for a linear regression model are given.

Values above 100% in several of the dietary groups suggest that this fatty acid, in addition to being efficiently stored in the fish, was also produced to some extent from other fatty acids, most likely from 18:1n-9, which was retained in the fish to a much lower extent. This suggests selective beta-oxidation and/or bioconversion to other fatty acids. Despite an increased storage/production of erucic acid when the dietary content was low, the absolute amount of erucic acid in the salmon fillet increased with increasing dietary content of this fatty acid (see Figure 3).

**Figure 4:**
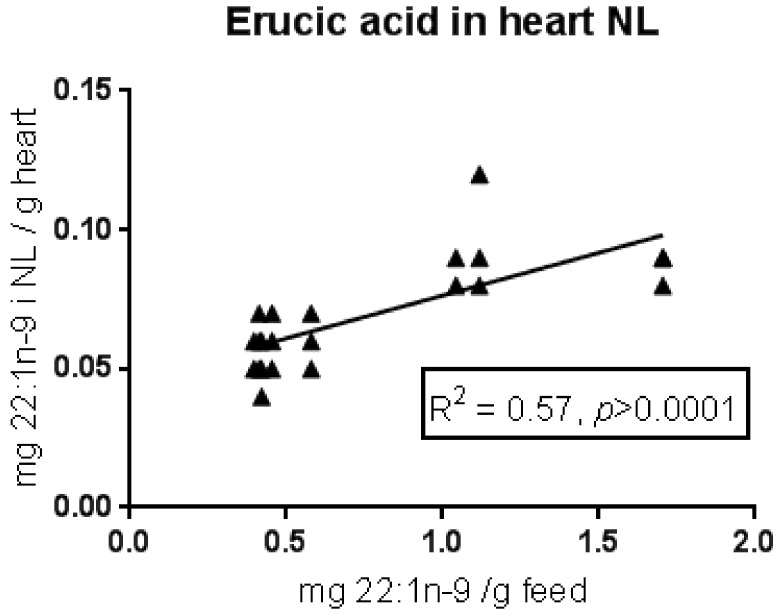
Content of erucic acid (22:1n-9) in neutral lipids (NL) of the heart in Atlantic salmon, in relation to the dietary content of erucic acid. The data are from a feeding trial [10] where salmon were given eight experimental feeds ranging from 40–170 mg erucic acid/100 g feed. The adjusted *R*-value and the *p*-value for a linear regression model are given.

In the polar lipids (cell membranes) of the heart, the content of erucic acid was mainly below the limit of detection (<0.1% of total fatty acid). This was also the case in the polar lipids of the liver. The content of erucic acid in liver neutral lipids (NL) was also low, but it increased somewhat with increasing dietary erucic acid (data not shown). Erucic acid was also low in neutral lipids in the heart, but somewhat higher than liver NL, and increased with increasing dietary erucic acid (Figure 4). There was no correlation between the total amount of neutral lipid in the heart and dietary erucic acid content (*p* = 0.26).

The feed with the highest erucic acid concentration in this trial contained 170 mg/100 g and it was a feed with a high inclusion of low erucic acid rapeseed (canola) oil (18% of the recipe/74% of added oil), while the use of other vegetable oils gave lower concentrations in the other feeds.

### 3.3. Erucic Acid in Seafood

**Figure 5:**
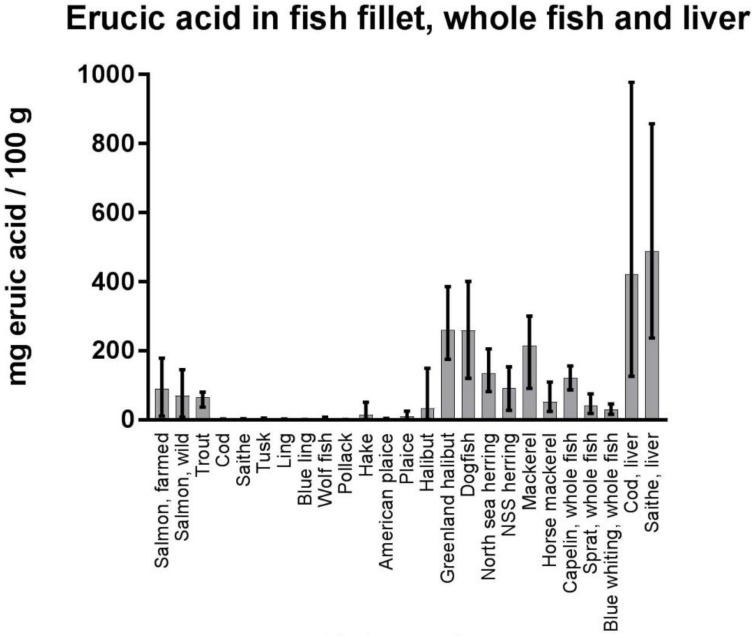
Content of erucic acid (22:1n-9), given as mg fatty acid/100 g sample in fish (in fillets if not otherwise specified, as well as some data for whole fish and fish livers towards the right of the figure). The bars show the mean values of erucic acid, as well as the minimum and maximum values. The number of samples analyzed for each category (N) are as follows; salmon, farmed *n* = 590, salmon, wild *n* = 99, trout *n* = 16, cod *n* = 60, saithe *n* = 40, tusk *n* = 63, ling *n* = 70, blue ling *n* = 10, wold fish *n* = 3, pollack *n* = 50, hake *n* = 46, American plaice *n* = 5, plaice *n* = 20, halibut *n* = 68, Greenland halibut *n* = 18, dogfish *n* = 15, North sea herring *n* = 19, NSS herring *n* = 30, mackerel *n* = 20, horse mackerel *n* = 21, capelin (whole fish) *n* = 10, sprat (whole fish) *n* = 8, blue whiting (whole fish) *n* = 10, cod liver *n* = 51, saithe liver *n* = 30. Many of the values were below the level of quantification (LOQ) for the analytical method, which is 1mg/100g. When some values were below the LOQ, 0 was used in calculation of the mean (lowerbound LOQ). Source of data: from the Institute of Marine Research’ seafood database [7].

Concentrations of erucic acid in fish fillet, some whole fish and fish livers are given in Figure 5.

**Figure 6:**
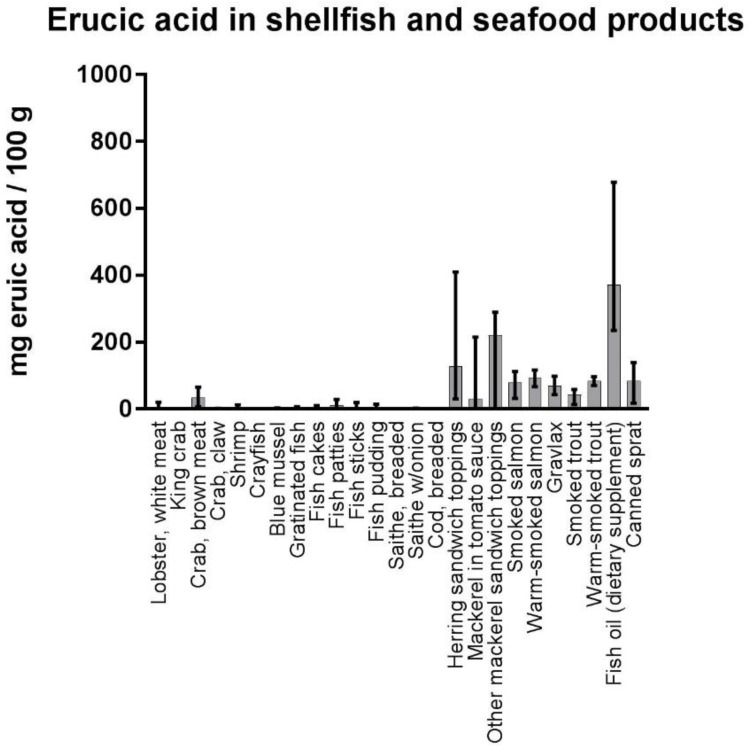
Content of erucic acid (mg fatty acid/100 g sample) in processed seafood products sampled in Norway. The bars show the mean value of erucic acid, as well as the minimum and maximum values. The number of samples for each category were as follows; lobster, white meat *n* = 15, king crab *n* = 151, crab, brown meat *n* = 10, crab, claw *n* = 10, shrimp *n* = 11, crayfish *n* = 52, blue mussel *n* = 12, gratinated fish *n* = 5, fish cakes *n* = 5, fish patties *n* = 5, fish sticks *n* = 5, fish pudding *n* = 5, breaded saithe *n* = 2, saithe w/onion *n* = 5, breaded cod *n* = 3, herring sandwich toppings *n* = 44, mackerel in tomato sauce *n* = 11, other mackerel sandwich toppings *n* = 13, smoked salmon *n* = 18, warm-smoked salmon *n* = 3, gravlax *n* = 6, smoked trout *n* = 4, fish oil (dietary supplement) *n* = 17, canned sprat *n* = 8.

The content of erucic acid was highly variable, among both species and individual samples within the same species. In farmed salmon, the mean concentration of erucic acid was 89.4 mg/100 g, with a range from 11.5 to 179.0 mg/100 g. Low levels were found in the fillets of lean fish species (e.g., cod, saithe, tusk, ling, wolf fish, and pollock, mean values from <LOQ–3.7 mg/100 g), as compared to the much higher levels in fillets of oily fish (e.g., mackerel, halibut, herring, and salmon, mean values from 33.8–260.4 mg/100 g). The highest levels were detected in liver of cod and saithe (mean values >400 mg/100 g). In processed seafood products (Figure 6), the highest concentrations of erucic acid were found in fish oil supplements for human consumption (mean value 371.3 mg/100 g and maximum value 678 mg/100 g), followed by mustard herring (mean value 334.4 mg/100 g) and different mackerel products (~250 mg/100 g in peppered mackerel, spiced mackerel, and cold- smoked mackerel).

Many of the values were below the level of quantification (LOQ) for the analytical method, which is 1 mg/100 g. When some values were below the LOQ, 0 was used in calculation of the mean (lowerbound LOQ). Source of the data: the Institute of Marine Research’ seafood database [7].

### 3.4. Human Consumption

The human intake of erucic acid from seafood depends on the amount of seafood consumed and the concentration of erucic acid in that seafood. Table 3 gives examples of erucic acid intake associated with consumption of a 200 g portion of different fish and the contribution to the TDI for a person with a body weight of 60 kg. A 200 g portion of farmed salmon with the mean analyzed concentration of erucic acid would contribute with 43% of the TDI in a 60 kg person, while 200 g of mackerel would cover the entire TDI (102%). However, at the maximum analyzed concentrations in these species, a 200 g portion of farmed salmon or mackerel would contribute 85% and 143% of the TDI, respectively.

## 4. Discussion

The ubiquitous presence of erucic acid in the lipids of fish and shellfish indicates that this fatty acid is naturally present in the marine food chain. Our data clearly show a relationship between the lipid content and the erucic acid content in various fish species, with a higher content in fish fillets with a high lipid level. These data support the limited data set on seafood from the EFSA report, showing the highest seafood levels of erucic acid in the oily fish species halibut, salmon, herring, sprat, and mackerel, with maximum values between 220–430 mg/100 g [4]. Average levels of erucic acid reported by the EFSA for salmon and trout’ samples (~100 mg/kg), were also comparable to those that were found in the present study. Higher concentration of erucic acid in farmed as compared to wild salmon probably reflect the higher lipid content in farmed salmon, which was reported to be almost twice as high in the farmed salmon [14]. Our data show that both fish oil and rapeseed oil are sources of erucic acid in commercial feeds for farmed salmon, contributing similar amounts. For fish oils, the geographical origin of the oil might affect erucic acid content, since long chain monounsaturated fatty acids are particularly high in oily fish species from the Northern hemisphere, such as capelin. However, the origin of the fish oils was not specified for the oils in the current dataset. In a previous salmon feeding trial that used capelin oil as the lipid source, the experimental feed contained 440 mg erucic acid/100 g feed [15], exceeding the levels of erucic acid in all of the commercial feeds that were analysed in the surveillance programme. This was gradually reduced to 120 mg/100 g with increasing replacement of the capelin oil by low erucic acid rapeseed oil (25–100% replacement). This shows that certain fish oils can be a greater source of erucic acid in fish feeds than rapeseed oil. However, high inclusion of fish oil is not a common feeding strategy used in commercial salmon feeds. Currently, commercial salmon feeds in Norway contain on average about two-thirds rapeseed oil (low erucic acid varieties) and one-third fish oil [16].

The main adverse effect of erucic acid found in various animal species is accumulation of lipid droplets (neutral lipids) in myocardial cells. This is a reversible effect, but high doses of erucic acid may cause degenerative lesions in the muscle fibers, followed by necrosis of the myocardial tissue [3]. A feeding study with high erucic acid rapeseed oil (HEAR) was conducted in Atlantic salmon [17], where both HEAR, low erucic acid rapeseed oil (LEAR), and soybean oil were used to replace capelin oil, resulting in dietary erucic acid concentrations varying from 0.3 to 28.1% of total fatty acids. There was no effect on weight gain, unlike observations in warm-blooded animals, possibly indicating a higher tolerance for this fatty acids in fish [17]. The lack of significant differences in heart lipid content after two and four weeks of feeding was probably due to low n and high variation (as there were large numerical differences), while heart lesions were not investigated. Heart lesions have been reported in Atlantic salmon fed sunflower oil [18], compared to few such lesions in fish fed a fish oil diet. While dietary erucic acid level was not reported in that paper, it is likely to have been higher in the fish oil diet. Consequently, the dietary content of erucic acid was not a likely cause of these lesions. The presence of lesions in the coronary artery have been investigated in both a short term study in salmon fed with diets containing fish oil, rapeseed oil, or 50:50 fish oil and rapeseed oil [19], and in a long-term study where fish oil was compared with a blend of vegetable oils [20]. There were no apparent effects on lesions in the coronary artery of fish oil versus vegetable oil fed fish in either study. In the feeding trial described in the current paper, there was no correlation between the total amount of neutral lipid in the heart and dietary erucic acid content, and hence no indication that erucic acid causes an accumulation of neutral lipids in the heart at these low dietary levels. In another study, increased level of TAG was found in the heart of salmon fed vegetable oil when compared to salmon fed fish oil, while the content of erucic acid in both heart tissue and muscle tissue was the same in both dietary groups (erucic acid levels were not reported for the diets) [21]. To sum up, there is limited information on how erucic acid might affect lipid accumulation in the heart, development of lesions or other health parameters in farmed fish, but the available information does not indicate such effects at the concentrations of erucic acid present in commercial salmon feeds.

Salmon and other fish are capable of both chain elongation and beta-oxidation of fatty acids, and are hence able to produce erucic acid from shorter chain fatty acids, such as oleic acid (18:1n-9) and gondoic acid (20:1n-9), as well as from longer chain fatty acids, such as nevronic acid (24:1n-9). Fish are also able to desaturate fatty acids, including the insertion of double bonds in the n-9 position of the carbon chain, and are thus theoretically able to produce erucic acid from saturated fatty acids. Although the retention of erucic acid (possibly including some in vivo production) appeared to increase with decreasing dietary levels in the present feeding trial, total erucic acid levels in the fish decreased with decreasing dietary levels. The retention of this fatty acid is also likely to be dependent on feed concentrations, as most other fatty acids are retained in salmon to a high extent when low levels are provided in the feed (like for erucic acid in the present trial), while the retention is much lower when feed concentrations are high [22]. In support of this, fish that were fed a HEAR diet (with erucic acid constituting 28.1% of dietary fatty acids) for 18 weeks, had only 6.5% erucic acid of the total fatty acids in heart triacylglycerols and 11.5% in muscle triacylglycerols [17]. If one would want to reduce the amount of erucic acid in farmed fish as compared to current levels, this could easily be achieved by utilizing other lipid sources than fish oils and rapeseed oil, for instance, other vegetable oils.

Our data indicate that seafood consumption might contribute considerably to the TDI, especially for population groups with a high intake of oily fish. One may even exceed the TDI by eating a single 200 g portion of mackerel. Based on the maximum erucic acid concentration found in cod liver, a 50 g serving would exceed the TDI for a 60 kg person. However, it must be taken into account that the TDI is a chronic health-based guidance value and includes an uncertainty factor of 100. It is more correct to compare the TDI with chronic exposure rather than intake from a single meal. In the EFSA report, the contribution of “fish meat” to total erucic acid exposure was important in some adult populations in different dietary surveys, with contributions up to 41% of the total exposure [4]. Higher contributions than this could be expected in certain population groups in Norway (and high consumption population groups in other European countries), due to the consumption of oily fish, fish liver, and the intake of fish oil supplements, such as cod liver oil. In Norway, the mean consumption of fish in two-year old’s is 16 g/day, and the 95th percentile consumption is 36 g/day; for adults, the mean consumption of fish is 52 g/day and the 95th percentile consumption is 201 g fish /day [23]. Mean consumption of fish oils and cod liver oil in two-year-old’s was 2 g/day, while the 95th percentile was 6 g/day; in adults, the mean consumption of fish oils and cod liver oil was 3 g/day and the 95th percentile was 10 g/day [23]. However, these data are not detailed enough to regarding distribution of fish species etc to assess erucic acid exposure, which should be included in future risk-benefit assessments of seafood.

Despite the potentially high contribution of oily fish to erucic acid intake, there are few indications that seafood intake, and, in particular, oily fish, has a negative impact on cardio-vascular health in humans. On the contrary, the risk of cardiovascular disease was reported to be reduced by the intake of oily fish, see, e.g. [24]. The Norwegian Scientific Committee for Food Safety (VKM) has conducted risk-benefit evaluations of fish consumption in the Norwegian diet and recommends that most people should increase their consumption due to the health benefits [23]. However, erucic acid exposure has not been specifically addressed in these risk-benefit evaluations. Increased levels of 22:1 fatty acids in plasma phospholipids have been associated with a higher incidence of congestive heart failure in two independent cohorts [25]. However, fish consumption seemed to lower the risk of congestive heart failure in both of these cohorts [26,27]. In another cohort, higher levels of erucic acid in erythrocytes was associated with lower incidence of coronary heart disease, leading the authors to conclude on a cardioprotective effect [28]. Another study on patients with coronary artery disease found no association between erucic acid in erythrocytes and all-cause mortality or CVD-mortality [29]. Indian studies have shown positive effects of mustard seed oil, which is high in erucic acid, on ischemic heart disease, and myocardial infarction [30,31]. These positive effects are more likely to be associated with the high content of α-linolenic acid (18:3n-3) in mustard seed oil in a population with a generally low seafood intake, but at least they occurred despite the high content of erucic acid.

There are currently a number of knowledge gaps regarding erucic acid, and especially erucic acid from seafood. Data on health effects of erucic acid in humans is scarce, and the TDI set by EFSA is based on effects that were observed in animal studies [4]. Exposure should be assessed in population groups with a high consumption of oily fish and/or fish oil, and erucic acid should be specifically included in future risk-benefit assessments regarding seafood intake.

## 5. Conclusions

The concentrations of erucic acid in fish and other seafood varies considerably among species and individual samples, with the highest concentrations being found in fish liver, fish oil, and in the fillet of oily fish. Consumption of a 200 g portion of oily fish contributes significantly to the TDI set by EFSA in 2016, and may even exceed it.

There are currently many knowledge gaps regarding erucic acid, and further studies regarding its metabolism and its health effects in fish and humans are warranted. This paper contributes with new knowledge on the presence of erucic acid fish feeds, fish, and seafood products.

## Figures and Tables

**Table 1:** Erucic acid (22:1n-9) as percentage of total fatty acids in fish feed and feed ingredients. The table shows the number of samples (N) for each year and matrix, the mean value of erucic acid, as well as the minimum and maximum values.

Year	Matrix	N	Mean	Min.	Max.
2004		1	1.0	1.0	1.0
2005		6	0.9	0.7	1.1
2006	Fish silage	8	1.1	1.0	1.2
2007		5	0.9	0.5	1.3
2008		2	1.0	1.0	1.0
2007		1	0.1	0.1	0.1
2008	Fish meal	4	0.6	0.1	1.3
2004		2	0.1	0.1	0.2
2005	Vegetable oil	3	0.8	0.2	1.5
2006	(uspecified)	4	0.2	0.0	0.5
2007		2	0.8	0.7	0.8
2008		1	0.7	0.7	0.7
2004		3	0.6	0.2	0.8
2005	Rapeseed oil	9	0.8	0.2	2.4
2006	(canola)	7	1.3	0.0	3.2
2007		7	0.7	0.3	0.9
2008		7	0.7	0.2	2.7
2004		6	1.4	0.1	2.4
2005	Fish oil	10	0.6	0.1	1.8
2006		9	0.7	0.1	2.1
2007		10	0.7	0.2	1.5
2008		6	0.7	0.1	1.1
2006		30	0.8	0.0	1.7
2008		10	0.8	0.3	1.0
2012	Fish feed	13	0.6	0.2	1.1
2013		69	0.5	0.2	1.0
2014		73	0.5	0.2	0.9
2015		20	0.4	0.2	0.9
2016 ^10^		20	0.3	0.2	0.5

^10^, [13]. Data from the other years have not been published in reports.

**Table 2:** Concentration of erucic acid (22:1n-9), given as mg fatty acid/100 g sample, in fish feed. The table shows the number of samples (N) for each year, the mean value of erucic acid, as well as the minimum and maximum values.

Year	N	Mean	Min.	Max.
**Fish Feed**				
2012	13	152	29	317
2013	69	138	22	250
2014	73	146	56	333
2015	20	113	53	181
2016 ^5^	20	096	52	151

^5^, [13].

**Table 3:** Intake of erucic acid (22:1n-9) when consuming 200 g fish and the proportion of the Tolerable Daily Intake (TDI) of 7 mg per kg bodyweight per day, for a 60 kg person, using the mean and the maximum value for the respective fish species.

Species	Mean Value mg Erucic Acid	% TDI	Max Value mg Erucic Acid	% TDI
Farmed salmon	179	43	358	85
Wild salmon	141	34	290	69
North sea herring	271	65	411	98
Mackerel	429	102	601	143
